# Paradoxical embolism following thromboaspiration of an arteriovenous fistula thrombosis: a case report

**DOI:** 10.1186/1752-1947-4-345

**Published:** 2010-10-28

**Authors:** Bouteina Bentaarit, Anne Marie Duval, Anne Maraval, Djamal Dahmane, Karine Dahan, Brahim Amara, Philippe Lang, Djillali Sahali

**Affiliations:** 1 Service de Néphrologie, AP-HP, Groupe hospitalier Henri Mondor - Albert Chenevier, Créteil, F-94010 France; 2 Service de neuroradiologie, AP-HP, Groupe hospitalier Henri Mondor - Albert Chenevier, Créteil, F-94010 France; 3 Service de cardiologie, AP-HP, Groupe hospitalier Henri Mondor - Albert Chenevier, Créteil, F-94010 France

## Abstract

**Introduction:**

Paradoxical embolism is an increasingly reported cause of arterial embolism. Several embolic sources have been described, but thrombosis of an arteriovenous fistula as a paradoxical emboligenic source has not, to the best of our knowledge, been reported.

**Case presentation:**

A 50-year-old Caucasian woman received a renal graft for primary hyperoxaluria. After transplantation, she was maintained on daily hemodialysis. Thrombosis of her arteriovenous fistula occurred two weeks post-transplantation and was treated by thromboaspiration, which was partially successful. During a hemodialysis session immediately following thromboaspiration, she developed a coma with tetraplegia requiring intensive cardiorespiratory resuscitation. Brain magnetic resonance imaging revealed various hyperdense areas in the vertebrobasilar territory resulting from bilateral occlusion of posterior cerebral arteries. Transesophageal echocardiographic examination showed a patent foramen ovale, while pulse echography of the arteriovenous fistula revealed the persistence of extensive clots that were probably the embolic source. A paradoxical embolus through a patent foramen ovale was suggested because of the proximity of the neurological event to the thrombectomy procedure.

**Conclusions:**

The risk of paradoxical embolism in a hemodialyzed patient with a patent foramen ovale deserves consideration and requires careful evaluation in situations of arteriovenous fistula thrombosis.

## Introduction

The foramen ovale is usually obliterated following the establishment of adult circulation, but remains patent in 25% of individuals [1]. This potential communication between the venous and arterial circulation can allow thromboembolic material to bypass the lungs and enter the systemic circulation. Paradoxical embolism occurs when a thrombus from the venous circulation passes into the arterial circulation through an intracardiac or vascular defect. We describe a case of paradoxical embolization through a patent foramen ovale (PFO) following thromboaspiration for acute thrombosis of an arteriovenous (AV) fistula. Although the risk of pulmonary embolism in this setting is well documented [2], to the best of our knowledge, no paradoxical embolism after thromboaspiration has been reported.

## Case presentation

A 50-year-old Caucasian woman was admitted for kidney transplantation. Renal history began at 10 years of age when she was hospitalized for several episodes of ureteral colic due to recurrent stone disease. She was married with four children. During each pregnancy, she was treated for hypertension. At 40 years of age, she was evaluated for severe arterial hypertension. Investigations revealed proteinuria (2 gr/24 h) and an increase in plasma creatinine (12 μmol/dL). Renal ultrasound showed multiple stones in the left kidney and bladder, while the right kidney was atrophied. The diagnosis of primary hyperoxaluria type I was established by the detection of the G170R mutation in the peroxisomal alanine-glyoxylate aminotransferase (AGT) gene. The residual activity of AGT was less than 28% in biopsied liver tissue from our patient. As chronic renal failure progressed, our patient experienced, at 44 years of age, multiple embolic episodes involving the femoral arteries, hypogastric arteries, splenic artery and renal arteries, and requiring surgical thrombectomy. Transthoracic echocardiography revealed an intraventricular thrombus associated with hypokinesia of the apical region and the interventricular septum. Hematological studies including tests for thrombophilia (protein C and protein S deficiency, factor V Leiden, anti-thrombin III deficiency, and anti-phospholipid antibody syndrome) and serum homocysteine concentrations yielded normal results. An electrocardiogram did not reveal any signs of atrial fibrillation or myocardial infarction.

**Figure 1:**
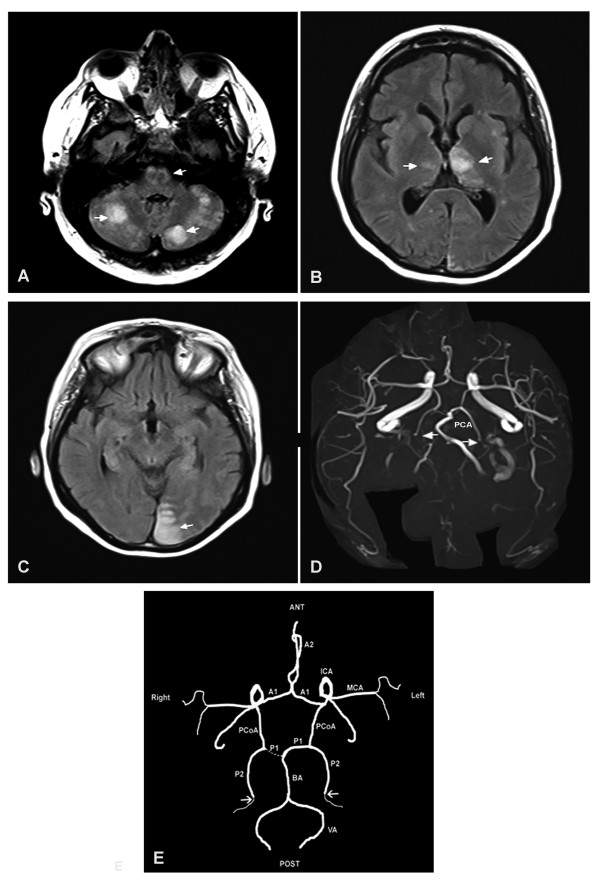
**Acute vertebrobasilar territory stroke following percutaneous thromboaspiration of an occluded arteriovenous fistula of the forearm**. **(A) **Magnetic resonance imaging (MRI) of the head. Serial 5 mm axial brain images show the presence of bilateral hypersignal areas (indicated by arrowheads) in the cerebellar hemispheres and bulboprotuberancial junction. **(B) **Hypersignal in the thalamic areas, predominantly on the left. **(C) **Hypersignal in the occipital lobe. **(D) **Polygon of Willis by MRI time of flight show bilateral occlusion of the posterior cerebral arteries. **(E) **Polygon configuration of Willis in our patient. The arrows show the localization of bilateral occlusion of the Posterior cerebral arteries (PcoA, posterior communicating artery; PCA, posterior cerebral artery (P1 and P2 indicate the PCA segments); ACA, anterior cerebral artery (A1+A2 indicate the ACA segments); MCA, middle cerebral artery; ICA, internal carotid artery; BA, basilar artery; VA, vertebral artery. Note that our patient displays a "fetal type" right PCoA, in that the P1 segment is hypoplastic and the ICA supplies right posterior cerebral territory via PCoA.

Our patient was discharged with long-term treatment with anti-vitamin K. Terminal renal failure was precipitated by bilateral renal artery embolism requiring the initiation of hemodialysis, which was performed five days per week because of hyperoxaluria. Kidney transplantation was performed two years following the initiation of dialysis, but the graft was rapidly lost at day seven post-transplantation, secondary to thrombosis of the graft vein. Our patient was maintained on periodic hemodialysis for four years, after which she received a second renal transplantation from a cadaveric donor. The immediate course was marked by delayed graft function allowing the deferred use of calcineurin inhibitors. The immunosuppressive regimen consisted of anti-thymocyte globulin (ATG) and methylprednisolone therapies. Heparin therapy was started 12 hours following the surgical procedure. A kidney biopsy on day 14 post-transplantation revealed the presence of calcium oxalate crystals in the parenchyma, which led to the start of daily dialysis using a large surface membrane. Diuresis progressively recovered from day 18 post-transplantation but dialysis was continued. On day 22, our patient developed major bleeding with acute anemia, requiring blood transfusion and the termination of heparin therapy. A non-infusion computed tomography (CT) scan of the abdomen revealed a perigraft hematoma. Three days later (on day 25), she developed thrombosis of her AV fistula. Anti-coagulation treatment with danaparoid sodium (Orgaran) was reinitiated. A tunneled silicone catheter was inserted in the right femoral vein. A revascularization procedure by angioplasty and stent deployment was attempted five days later. A 6 French vascular sheath was placed upstream of the occluded venous arm of the fistula, then replaced by a 9 French vascular sheath to increase the lumen of the occluded vein, after which a 6 French catheter was used to aspirate the clots. However, although the fistula was only partially repermeabilized on the side of the venous anastomosis, it was immediately reused for hemodialysis. While our patient was alert and her status was normal on initiation of the dialysis session (Glasgow Coma Scale 15), three hours later she developed diffuse seizures and became rapidly unresponsive (Glasgow Coma Scale 3), prompting intubation for airway protection. Magnetic resonance imaging showed multiple areas of increased density representing severe ischemia in the vertebrobasilar territory, which result from bilateral occlusion of posterior cerebral arteries (Figure 1).

**Figure 2:**
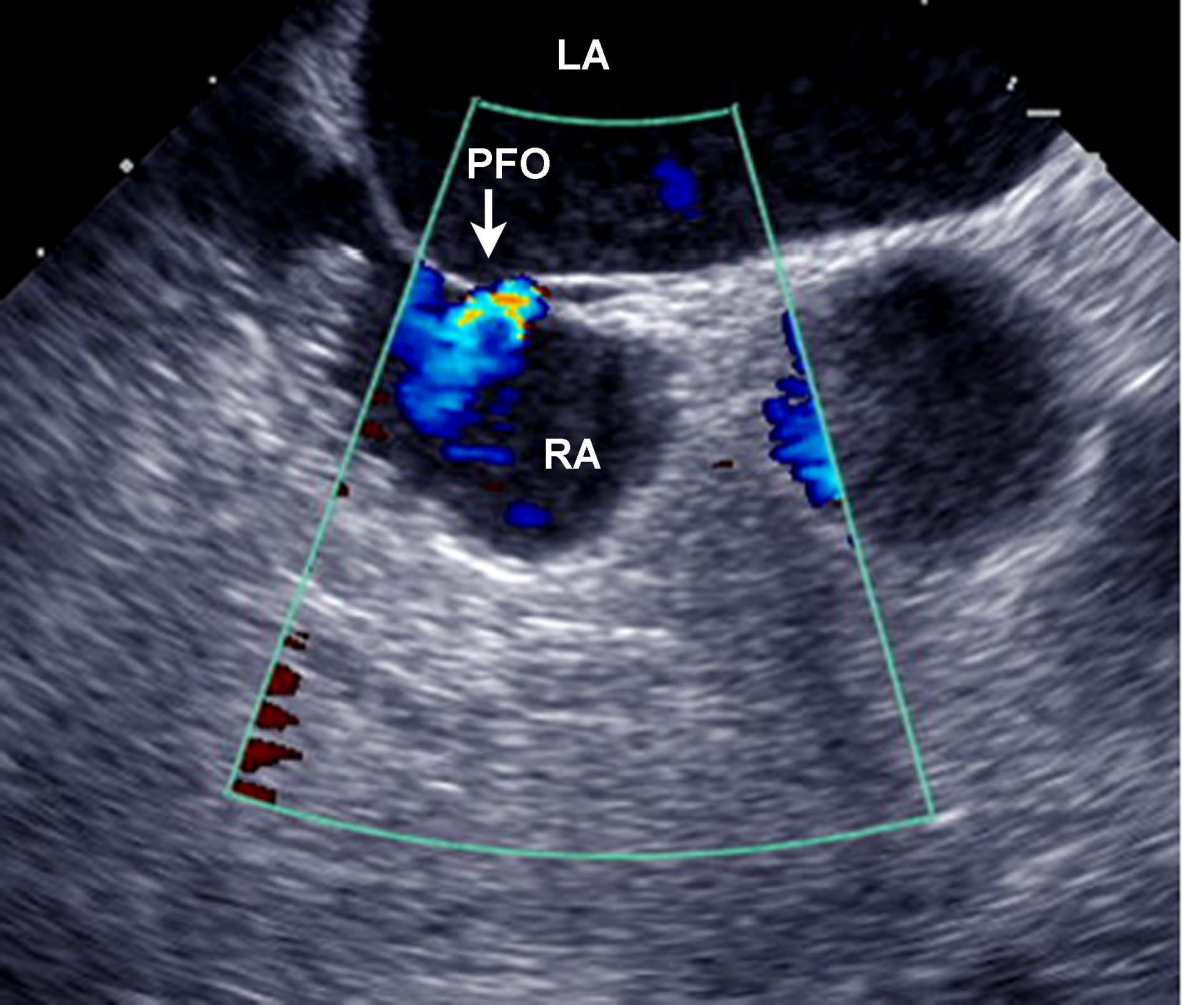
**Transesophageal echocardiography**. A patent foramen ovale (PFO) with a left-to-right shunt is shown at the level of the interatrial septum. RA and LA indicate right and left atria.

Our patient was managed in our intensive care department. Transesophageal echocardiography (TEE) performed two months later revealed a previously unknown PFO with a left-to-right shunt at the interatrial septum (Figure 2). However, contrast echocardiography test was not performed because of the absence of permeable peripheral veins in our patient. The TEE examination did not reveal other embolic sources in the ascending aorta and aortic arch.

Our patient was discharged from the intensive care department with stable renal function (serum creatinine at 15 μmol/dL) two months after admission. The serum oxalate concentration was 2.15 μmol/dL (normal: below 33 μmol/dL). Neurological evaluation six months after the stroke showed the persistence of weakness predominantly in the inferior limbs, as well as dysarthria, anterograde amnesia and hyperemotivity.

## Discussion

The number of patients maintained on periodic hemodialysis is increasing due to the improvement of the long term survival of this patient population and because of an exponential increase of diabetic kidney disease. Currently, the management of AV fistula thrombosis is essentially based on thromboaspiration performed by an interventional radiologist. This technique is commonly endowed with a high rate of success and a low rate of complications [3].

Pulmonary embolisms as well as fatal paradoxical embolism of the brain after percutaneous thrombolysis of a hemodialysis graft have been reported [2]. These complications remain rare, as shown by the finding that in a large series totaling 1460 cases treated by mechanical thrombolysis, no symptomatic pulmonary or systemic embolisms occurred [4]. Nonetheless, the morbidity and mortality rates are more significant in patients with paradoxical embolism [2].

The clinical diagnosis of paradoxical embolism requires the presence of deep venous thrombosis and/or pulmonary embolism, an intracardiac defect with a right-to-left shunt, associated with the absence of an emboligenic source in the left side of the heart. In our patient, the timing of the paradoxical embolism, which occurred after a thromboaspiration procedure for AV fistula thrombosis, the absence of an arterial emboligenic source and the documentation of a PFO with left-to-right shunt by TEE strongly suggest that the brain emboli arose from the thrombosed AV fistula. The severity of the paradoxical embolism made it impossible to perform contrast or bubble echocardiography to visualize the inversion of the shunt in the early phase of the stroke. Paradoxical embolism results from the inversion of the shunt, caused by an acute increase in right atrial pressure, in our case a possible direct consequence of the thromboaspiration procedure through venous migration of the clots or hemodynamic changes. When right atrial pressure exceeds that of the left, the inversion of the shunt (right-to-left) occurs through the PFO. More rarely, an acute elevation of right atrial pressure can occur in the setting of chronic pulmonary heart disease or Valsalva maneuver. In our patient, pulse echography did not detect any vein thrombosis, except that of the AV fistula. Paradoxical embolism is frequently associated with pulmonary embolism, which is a common cause of increased right atrial pressure.

Given that the prevalence of PFO is 25% in the general population, the possibility of a paradoxical embolism must be suspected in patients who present with arterial embolism without a clearly identified cause such as atrial fibrillation, carotid artery stenosis or cholesterol embolism. A PFO has been diagnosed in 50% of patients with stroke compared with 15% of control subjects [5]. The most frequent sites of paradoxical embolism are the extremities (50%) and the brain (40%), while the heart, spleen and kidney are more rarely affected [6].

In this case, multiple emboli occurring while our patient displayed moderate renal failure probably arose from the intraventricular thrombus revealed by transthoracic echocardiography. In retrospect, it is possible that this mural thrombus may have masked a pre-existing PFO. A paradoxical embolism revealing a vermicular thrombus trapped in the PFO with floating parts in the right and left atrium, complicating a deep-vein thrombosis of the right superficial femoral vein, has recently been reported in an 85-year-old woman [7]. Paradoxical embolism involving multiple organs has also been reported [8].

The diagnosis of PFO is best accomplished by contrast or bubble TEE because it more accurately reveals PFOs with a right-to-left atrial shunt. Its sensitivity and its specificity approach 96% [9].

The treatment of paradoxical embolism includes anti-platelet agents, systemic anticoagulation and closure of the PFO [10]. In its acute phase, paradoxical embolism is usually treated by thrombolytic and heparin therapy, followed by anti-vitamin-K therapy, which should be continued for three to six months. Anti-vitamin-K therapy should be continued indefinitely in the following situations: (i) recurrent paradoxical embolism; (ii) persistent PFO; (iii) chronic obstructive pulmonary disease-induced pulmonary hypertension that leads to increased right atrial pressure and shunting through the PFO. Given the prevalence of PFO in the general population, closure is indicated only in some pathological situations such as recurrent paradoxical embolism or when systemic anti-coagulation is contraindicated. Closure can be accomplished by percutaneous or surgical methods.

## Conclusions

The severity and potential fatal outcome of paradoxical embolism raises the question of PFO screening in patients treated by hemodialysis. When a patient with a PFO with a known right-to-left shunt develops thrombosis of an AV fistula, it is advisable for the interventional radiologist and the surgeon to choose the technique that presents the lowest risk of embolization, while informing the patient of the potential risk.

## Competing interests

The authors declare that they have no competing interests.

## Consent

Written informed consent was obtained from the patient for publication of this case report and any accompanying images. A copy of the written consent is available for review by the journal's Editor-in-Chief.

## Authors' contributions

BB, AMD, AM, DD, KD and BA managed the patient in their respective departments and contribute to the preparation of this case report. PL is the supervisor of the management and DS has managed the patient and written this case report. All authors read and approved the final manuscript.
